# Managing Free-Range Laying Hens—Part A: Frequent and Non-Frequent Range Users Differ in Laying Performance but Not Egg Quality

**DOI:** 10.3390/ani10060991

**Published:** 2020-06-06

**Authors:** Terence Zimazile Sibanda, Manisha Kolakshyapati, Mitchell Welch, Derek Schneider, Johan Boshoff, Isabelle Ruhnke

**Affiliations:** 1 School of Environmental and Rural Science, Faculty of Science, Agriculture, Business and Law, University of New England, Armidale NSW 2351, Australia; mkolaks2@une.edu.au (M.K.); iruhnke@une.edu.au (I.R.); 2 Precision Agriculture Research Group, Faculty of Science, Agriculture, Business and Law, School of Science and Technology, University of New England, Armidale NSW 2351, Australia; mwelch8@une.edu.au (M.W.); dschnei5@une.edu.au (D.S.); 3 Computation, Analytics, Software and Informatics, University of New England, Armidale NSW 2351, Australia; jboshoff@une.edu.au

**Keywords:** behaviour, economics, efficiency, husbandry, movement, non-caged, poultry, production, Radio-Frequency Identification (RFID), sub-populations

## Abstract

**Simple Summary:**

Free-range laying hens are allowed to roam around and exhibit their desired behaviours including usage of the outdoor range. Consequently, flock sub-populations of high-range users (“rangers”) and low-range users (“stayers”) appear. The subpopulation effect on egg production and egg quality has not been studied to date. Rangers were more consistent in their range use, while stayers increased their range use over time. Rangers came into lay earlier compared to the stayers but their egg production dropped during mid-lay until the end of lay when they were outperformed by the stayers. The flock sub-populations did not differ in their egg quality during the production period. These findings are of relevance to egg producers, as it allows them to manage their flock knowing that they can encourage them to range/stay in the shed without compromising egg quality, including off-grade eggs. In the future, it would be interesting to investigate if increased egg performance was triggered by the more frequent range usage.

**Abstract:**

Little is known about the impact of ranging on laying performance and egg quality of free-range hens. The aim of this study was to characterise egg production of commercial free-range laying hen sub-populations of low-, moderate- and high-range use at an early age. A total of five flocks with 40,000 hens/flock were investigated where 1875 hens/flock were randomly selected at 16 weeks of age, monitored for their range use and subsequently grouped into “stayers” (the 20% of hens that spent the least time on the range), “roamers” (the 20% of the hens that used the range more than stayers but less than rangers) and “rangers” (the 60% of the hens that spent the most time on the range). Eggs from the individual groups were collected in 10-weekly intervals until hens were 72 weeks of age, commercially graded and tested for several quality parameters. Significant differences were noted for hen-day production. For example, at 22 weeks of age, rangers enjoyed a laying rate of 88.0% ± 1.1%, while stayers performed at 78.2% ± 1.9% but at 72 weeks of age egg production of rangers was 85.1% ± 0.9% and of stayers was 95.5% ± 0.9% (*p* < 0.05). Range use was of minor importance to the egg quality.

## 1. Introduction

Optimising egg production is one of the main goals of the industry to increase the efficiency and ultimately, the sustainability of the free-range system. Egg quantity and quality are some of the most important traits of laying hens as they directly affect the economic benefits of poultry farmers [1]. Many research studies and literature reports have documented the reasons for reduced egg performance and inadequate egg quality [2,3,4]. The majority of challenges apply to the free-range egg industry, where a larger range of factors can compromise hen performance and, subsequently, farm economics. Aviary systems in cage-free facilities are designed to improve animal welfare by providing various features such as vertical space using perches and tiers, but can also provide horizontal space in form of winter gardens or, in case of free-range housing, ranges [5,6,7]. Previous studies revealed that a proportion of birds never leave the hen house, while others spend the majority of time on the range [8,9,10]. The consequences of these flock sub-populations can include reduced flock uniformity, sub-optimal flock nutrition, and (potentially) sub-optimal egg production [11,12,13]. There is no doubt that uniform body weight is crucial to obtain the targeted egg performance and minimise loss [14,15]. However, hens in non-caged systems are not only at risk to exhibit lower flock body weight uniformity but also subject to non-uniform behaviour [9,10,12,16]. While egg production is highly influenced by hen genetics, diet, light exposure, pullet quality, and housing including nest box management, the impact and management decisions related to flock sub-populations have not been investigated in-depth [1,17]. For example, poor eggshell quality at the end of lay does not mean that all hens in an ageing flock produce eggs of reduced quality, rather the variability in egg quality within the flock increases. Common problems associated with cage-free systems include the production of overall fewer eggs, flocks not reaching the targeted breed standard weight, and a relatively high percentage of off-grade eggs. For example, the percentage of off-grade eggs that cannot be commercially sold as whole egg to the retail market typically ranges between 2% and 5%, with highest values up to 10% at the end of lay in free-range farming systems (personal observation). If egg quality and quantity can be maintained until the end of lay, the laying period can be extended, resulting in more economic and sustainable use of hens. Therefore, the aims of this study were to (1) describe ranging sub-populations of commercial free-range laying hens and to (2) investigate the effects of range use on egg production and egg quality.

## 2. Materials and Methods

### 2.1. Ethical Statement

All procedures carried out in this experiment were approved by the University of New England’s Animal Ethics Committee (AEC 16-087).

### 2.2. Animal Housing and Management

A total of 5 commercial free-range flocks (Flocks A–E) were investigated where ~1875 hens/flock were randomly selected, monitored for their range use, and then grouped according to their range use. In detail, the experimental hens (Lohmann Brown classic) were placed in the layer shed at 16 weeks of age amongst their peers of ~38,125 hens to compose a flock of ~40,000 hens each. All five flocks were placed in succession from December 2016 to June 2018 into their sheds of the same construction and with the same geographic orientation. At the day of placement, the experimental hens (5 × ~1875 = ~9375 hens) were individually marked using numbered and colour-coded leg bands (Monza R6 UHF RFID Tags, Impinj, Seattle, WA, USA) and randomly placed in one of three experimental sub-sections of the shed, where a transverse partitioning of the shed and the range allowed these hens to use the same infrastructure as the remaining hens of the flock, while at the same time, being monitored using a Radio-Frequency Identification (RFID) System. Details of the experimental set up are illustrated in Sibanda et al., 2019 [12]. Every hen had access to a three-tier aviary system (Natura Step, Big Dutchman, MI, USA), including chain feeders, nipple drinkers, nest boxes, and perches. Wood shaving litter was available inside the shed and hens were placed targeting an indoor and outdoor stocking density of 9 hens/m^2^ and 1500 hens/ha, respectively. Five percent of the outdoor range was covered with an artificial cloth and there was a various degree of pasture with very little to no cover close to the shed, and increasing growth from 10 m distance onwards. The range subdivision was of rectangular shape in a 90° angle to the pop hole, with all ranges placed parallel to each other. The width of each range was 3.6 m and overall range area for each group was 4167 m^2^.

### 2.3. Range Use Monitoring

Range usage of individual hens was tracked using a custom-made RFID system (Science and Engineering Workshop at the University of New England, Armidale, NSW, Australia) with further detail described in [12]. The highest signal strength of −37.4 dB was recorded at 25 mm from the antenna. At this distance, 100% of RFID tags were recorded and the system was still effective at reading the tags at a distance of 150 mm with 90% accuracy [18]. An accuracy of 89.7% was also observed when comparing the number of chickens that visited the outdoor in the video observations and the RFID system output data. Individually numbered leg bands contained an RFID microchip, allowing hens to be traced when entering or leaving the range. Two RFID antennae placed in parallel along the entire length of the pop holes (the pop hole width covered the entire length of the experimental partition) made tracing of hen movement directionality feasible. Range access was offered from 09:00 to 20:00 daily except for the days of extreme weather conditions such as temperatures above 36 °C or on vaccination/medication days. The received RFID data was processed using Speedway R420 RFID tag readers (Impinj, Seattle, WA, USA), Clear Stream RFID software (Portable Technology Solutions, Calverton, NY, USA) and R-studio statistical software (v1.1.453, RStudio, Inc.).

### 2.4. Range Use Sub-Populations

**Figure 1:**
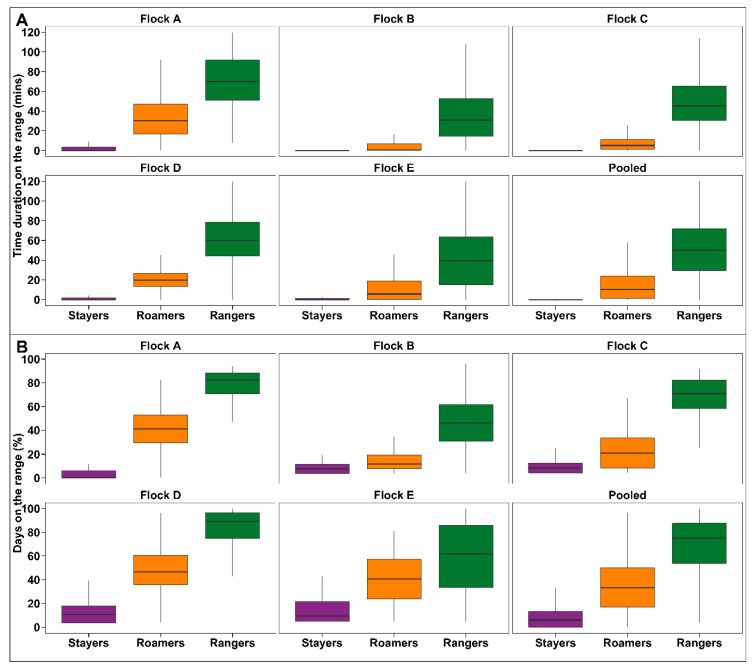
Box plot representing the identified ranging sub-populations of stayers (purple), roamers (orange), and rangers (green). The horizontal lines on the box plot represent the lower quartile, median and upper quartile. Figure 1A shows the mean time duration/hen/day of stayers, roamers and rangers on the range as determined for each of the five flocks investigated, as well as the pooled cohort. Figure 1B shows the percentage of days that the hens of the respective sub-population spent on the range. These results obtained when hens were between 18 and 21 weeks of age, were then used to classify and rearrange hens so individuals of the same category were placed together in their dedicated experimental shed subsection where they lived for the remaining laying period (until 74 weeks of age).

**Figure 2:**
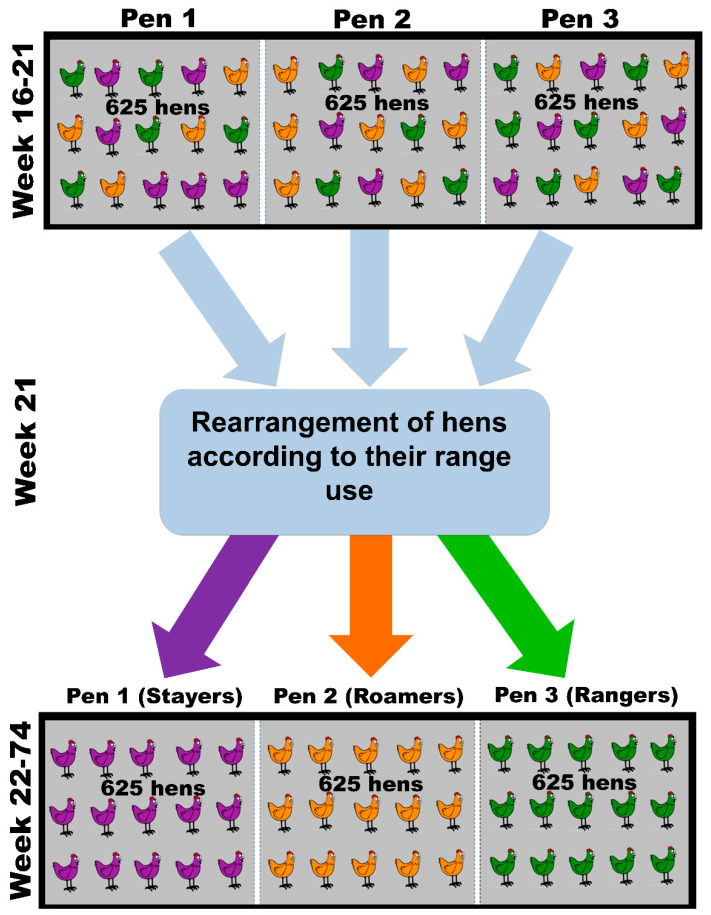
Rearrangement of hens according to their range use. The top part of the figure shows the composition of hens in the experimental unit sub-section after being placed when hens were 16–21 weeks of age. Because the hens were randomly selected, each sub-section contained hens that preferred to stay in the shed (“stayers”), but also hens that preferred to use the range every single day (“rangers”), or to explore occasionally (“roamers”). After the range use of these hens was monitored from 18–21 weeks of age, the hens were physically re-arranged to allow for the composition of flock sub-populations that consisted of stayers, roamers and rangers only (bottom part of the figure). Because these sub-populations were confined in the experimental sub-units (with access to the adjacent range), egg collection from these sub-populations was feasible.

The hens were individually weighed at 16, 22, and 74 weeks of age using poultry weighing scales (Veit, BAT 1, Moravany, Czech Republic) and the range use was monitored between 18–21 weeks of age to define the sub-populations. The range use recorded during this time was used to assign the experimental hens based on the number of days that the range was accessed. The experimental hens were then assigned to one of three sub-populations: the 20% of hens that spent the least time on the range were classified as “stayers”; the 20% of the hens that used the range more than stayers but less than rangers were classified as “roamers”; and the 60% of the hens that spent the most time on the range were classified as “rangers” (Figure 1). Although the rangers were 60% of the population, only 20% of the rangers’ sub-population were randomly selected to be used in this study and the remaining 40% were used in a different study, which allowed for equal number of hens for comparison in the present study. The average ± SEM time that individual hens of the stayers, roamers and rangers group spent on the range was 2.09 ± 0.15 min/hen/day, 18.3 ± 0.51 min/hen/day, and 55.6 ± 0.76 min/hen/day, respectively (Figure 1). Stayers, roamers and rangers visited the range on average 4.72% ± 0.31%, 40.6% ± 0.74% and 77.8% ± 0.56% of their available days. At the last day of week 21, hens were removed from their separate partitions and placed in cages for the duration of re-arranging. According to these group classifications, all individual hens of the subsections were physically rearranged into separate groups, to allow the composition of flock sub-populations that consisted of stayers (~625 hens), roamers (~625 hens) and rangers (~625 hens) only (Figure 2). Therefore, in each of the five commercial sheds used for this study, all stayers (5 × ~625 hens) were allocated to one experimental sub-section, all roamers (5 × ~625 hens) were allocated to a second experimental sub-section, and all rangers (5 × ~625 hens) were allocated to a third experimental sub-section. Due to the fact that each pen housed ~625 hens each, a comparable indoor stocking density of 9 hens/m^2^ was still given. The subsections were placed in the centre of each shed to avoid any effect of the end of the shed, not having hens ranging on both sides of the subsection, or similar. Individual range usage of all hens was then continuously monitored until hens were 72 weeks of age.

### 2.5. Egg Performance and Egg Quality

The egg production for each flock sub-population (stayers, roamers, rangers) was determined in 10 weekly intervals, when hens were 22, 32, 42, 52, 62 and 72 weeks of age. During these collection weeks, all eggs laid into the nest box were collected for the duration of seven consecutive days and subject to commercial on-farm grading. In addition, any eggs that were laid on the wire mesh of the aviary system were collected and recorded as system eggs, eggs laid on the litter and on the range were collected and recorded as floor eggs, and eggs with dirty shells or external shell defects (cracks, non-shelled eggs and misshapen eggs) were collected and recorded as waste. Records of these individual egg categories were maintained daily during the collection weeks and used for further analysis. For calculating egg production, the actual number of hens in each experimental sub-partition was determined at the beginning of each collection week by taking the mortalities that were observed to that date into account. The total number of eggs collected was then divided by the total number of hens present in the system, resulting in the daily laying percentage, which was then used for statistical analysis.

A subsection of the commercially graded eggs was used to determine the egg quality: Five eggs/pen/day were randomly selected from each pen for the duration of five consecutive days when hens were 22, 32, 42, 52, 62, 72 weeks of age. Individual egg weight, eggshell breaking strength, albumen height, Haugh unit and yolk colour was determined by using an egg multi-tester instrument (Nabel DET-6000, Kyoto, Japan).

### 2.6. Statistical Analyses

All the data were analysed using JMP Statistics software (v14 IBM SAS Institute Inc., Cary, NC, USA 1989–2007) except when stated otherwise. To describe the range use distributions within sub-populations, boxplots were created with summary statistics of average time duration and number of days on the range for all five flocks. To test for differences between sub-populations, the results from the sub-partitions were used as the experimental unit allowing for evaluation of five replicates after being investigated in five flocks (total *n* = 15). The effects of sub-population, hen age and their interaction with range usage, body weight, laying performance, egg quality and egg grade were analysed using a mixed restricted estimate of maximum-likelihood (REML) model with the flock as a random factor, and sub-population, hen age and their interactions as fixed effects. Following a significant main effect or interaction, Tukey’s HSD test was used to determine the significance of differences between means within that effect. For range use where unequal variances were observed, the Games Howell post-hoc test was carried out to compare the sub-populations using the ‘rstatix’ package of the R-studio statistical software (v1.1.453, RStudio, Inc.). A second series of descriptive statistics were presented as boxplot and violin plots to provide indications of distributions and variance for range use. To understand the trends of different measures, violin plots were overlaid with smooth spline fits of lambda 3.5. To show the difference between the sub-populations for range use uniformity, co-efficient of variation of the average time duration on the range of each sub-population were presented with a scatterplot and smooth spline fit (lambda 3.5).

## 3. Results

### 3.1. Range Use Differences of Sub-Population over Time

#### 3.1.1. Daily Mean Duration on the Range

**Figure 3:**
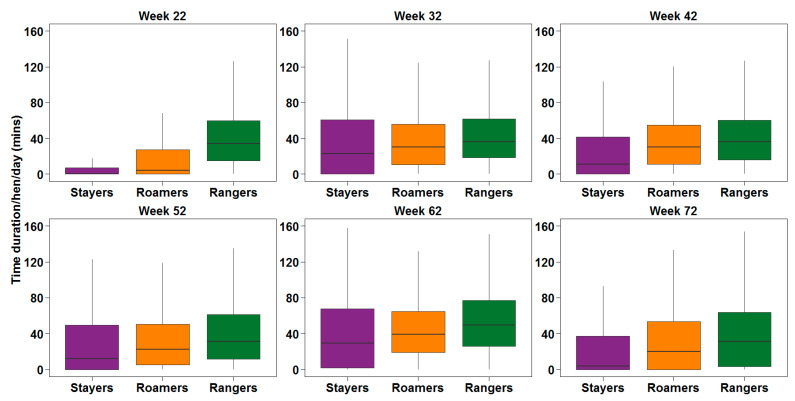
The average duration that the hens categorised as stayers (purple), roamers (orange), and rangers (green) spent on the range when the animals were 22, 32, 42, 52, 62 and 72 weeks of age. The data shown here are pooled from 5 flocks which served as replicates for statistical analysis.

**Figure 4:**
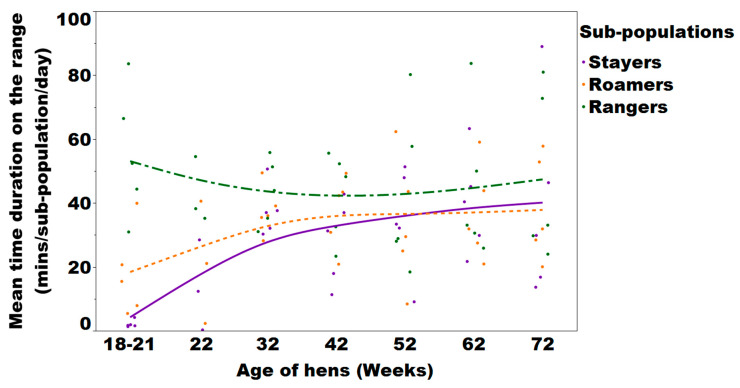
Trend lines and mean values that flock sub-populations spent on the range expressed as minutes/flock/day of the subpopulation of stayers, roamers, and rangers in Lohmann brown free-range laying hens during egg collection period of 22, 32, 42, 52, 62 and 72 weeks of age evaluated in 5 flocks. Each dot on the plot represents the average time duration that one sub-population replicate spent on the range.

Pooled flock descriptive statistics for the average time duration spent on the range by the individual hens and flocks are presented in Figure 3 and Figure 4. There was a significant sub-population and age effect on the daily mean duration on the range (*p* < 0.0001 and <0.0001, respectively). The Games Howell post-hoc analysis showed that daily mean range duration was higher for rangers compared to stayers and roamers (*p* < 0.0001) at every time point investigated or throughout the laying period. Similarly, the mean range duration/hen/day was always significantly less for stayers compared to rangers and roamers. There was also an interaction between sub-population and age of hens regarding the daily mean range use duration affecting stayers and roamers (*p* < 0.0001, Figure 4).

#### 3.1.2. Percentage of Days on the Range

**Figure 5:**
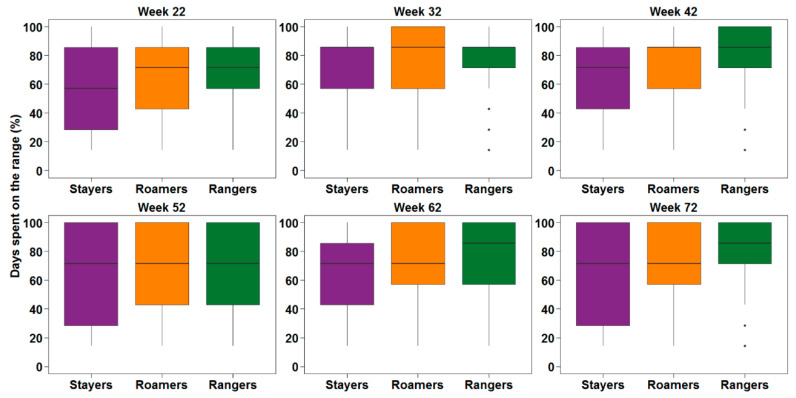
Boxplots showing the percentage days on the range of the sub-population of stayers (purple), roamers (orange), and rangers (green) when hens were 22, 32, 42, 52, 62 and 72 weeks of age. The data shown here are pooled from 5 flocks which served as replicates for statistical analysis.

**Figure 6:**
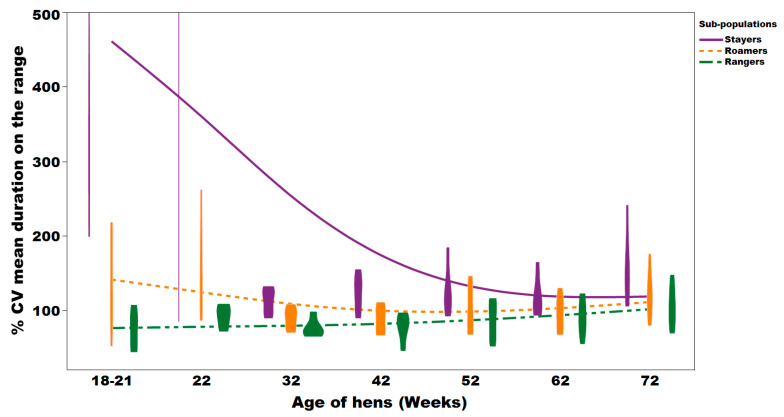
Individual variation of Lohmann brown free-range laying hens as shown by the% CV of average time duration on the range/day. A violin plot with an overlay of smooth spline (lambda = 3.5) representing the distribution and the% CV trend of stayers, roamers and rangers.

Pooled flock descriptive statistics for the percentage days with a range visit during the monitoring period for stayers, roamers and rangers are presented in Figure 5. A significant interaction between the subpopulation and the age of hens was found regarding the percentage of available days that hens spend on the range (*p* < 0.0001; Figure 6). A Games Howell post-hoc analysis showed that rangers spent a higher percentage of available days on the range compared to stayers and roamers (*p* < 0.0001). The difference between stayers and roamers was only significant for week 62.

### 3.2. Range Use Variation

The sub-population variation for the average duration spent on the range as shown by the coefficient of variation (CV), ranged between 91.0% and 500% with a mean of 137% ± 53.3% for stayers; ranged between 56% and 260% with a mean of 111% ± 8.09% for roamers and ranged between 45.6% and 145% with a mean of 85.7% ± 4.15% for rangers (Figure 6). There was an effect of sub-population, age of hens and interaction between sub-population and hen age (*p* = 0.0001; 0.0047; 0.0143, respectively; Figure 6). Overall, stayers had a higher CV compared to the roamers and rangers. The highest CV of 500% was observed for stayers at 22 weeks of age.

### 3.3. Bodyweight Differences of Flock Sub-Populations

The body weight difference of stayers, roamers and rangers are presented in Table 1. There was a significant main effects of sub-population, age of hens and their interaction on body weight (*p* = 0.0001). At 16 weeks of age, the rangers had a significantly higher body weight of 1.33 ± 0.021 kg compared to 1.30 ± 0.022 kg and 1.29 ± 0.021 kg of roamers and stayers, respectively (*p* = 0.0001). This difference was still maintained at 22 weeks of age with rangers, roamers and stayers weighing 1.78 ± 0.010 kg, 1.74 ± 0.008 kg, and 1.68 ± 0.012 kg, respectively, but not at 72 weeks of age.

### 3.4. Laying Performance and Egg Quality of Flock Sub-Populations

There was no overall difference between the sub-populations when investigating the laying performance of hens (*p* = 0.3634); however, there was an interaction between hen age and sub-population (*p* = 0.0001) where the laying performance of rangers decreased over time while that of stayers and roamers increased (Table 1). Rangers came into lay earlier compared to stayers and roamers. At 22 weeks of age, rangers had the highest laying rate of 88.0% ± 1.1%, while stayers performed at 78.2% ± 1.9% (*p* = 0.001). There was no significant difference between stayers and rangers at 32, 42, and 52 weeks of age, while the stayers outperformed the rangers with a margin of 4.5% and 10.4% at 62 and 72 weeks of age, respectively (Table 1).

### 3.5. Egg Quality Differences of Flock Sub-Populations

Albumen height did not differ between the sub-populations (*p* = 0.128) while albumen height decreased over time (*p* = 0.001; Table 2). There was a significant sub-population × hen age interaction of albumen height (*p* = 0.017): eggs obtained from rangers had the highest albumen height (7.40 ± 1.53 mm) at week 52 compared to eggs obtained from stayers and roamers (5.83 ± 0.17 mm and 5.61 ± 0.23 mm, respectively; Table 2). Egg weight did not differ between stayers, roamers and rangers at any time point, while it increased for all hens until 42 weeks of age after which it remained fairly constant (*p* = 0.001).

Yolk colour was graded on a scale 1 to 15, with 1 being brightest and 15 darkest. Yolk colour was not affected by range use (*p* = 0.4178) but decreased significantly over time until 62 weeks (*p* < 0.001; Table 2). The Haugh unit was significantly higher in stayers compared to rangers (*p* = 0.024; Table 2) and decreased in both groups significantly over time (*p* < 0.001; Table 2). Analysis of eggshell breaking strength revealed no significant effect between the sub-populations (*p* = 0.1995).

### 3.6. The Comparison of the Percentage of System, Floor and Waste Eggs

The percentage and proportion of system, floor and waste eggs collected from the sub-populations are presented in Table 3. There was a significant interaction between sub-populations and age of hens (*p* = 0.0177) for system eggs, where stayers laid less system eggs compared to rangers at 42 weeks of age. Stayers laid numerically more eggs in the system from 22 to 52 weeks of age, with numbers being twice as high for stayers compared to rangers (Table 3). In all sub-populations, the highest proportion of eggs laid on the system was at 22 weeks while the lowest systems were laid at 72 weeks of age, the overall decrease of system eggs over time was significant. While there was no significant effect of hen age on the floor eggs (*p* = 0.580), roamers laid overall significantly more floor eggs compared to stayers and rangers (*p* = 0.0148). The proportion of waste eggs increased significantly over time and reached its highest value of 0.35%; 0.57%; 0.50% for stayers, roamers and rangers at 72 weeks of age (*p* = 0.0001). Two interactions between hen age and flock sub-population could be observed, where roamers produced more waste eggs at 22 and 72 weeks of age but less or similar percentages at all other time points. However, the overall magnitude of that difference (at most 0.22%) was rather low. 

### 3.7. The Comparison of Flock Sub-Populations on Egg Grading

Age impacted egg size over time, with significantly larger eggs being produced with increasing hen age (*p* = 0.0001; Table 4). Interactions between sub-population and age could be observed for large, medium and economy eggs, where stayers and roamers laid significantly more economy and medium-sized eggs compared to rangers at 22 weeks of age to the expense of large eggs. However, this effect was not ongoing and comparable egg size were laid by all of these sub-populations thereafter.

Although there was no significant difference between sub-population on pulp eggs, there was an observed effect of age on the pulp eggs with the highest percentage of pulp eggs produced at 72 weeks of age (*p* = 0.0001).

## 4. Discussion

Free-range flocks have been commonly described as performing poorly compared to barn and caged hens [19,20]. However, the current results lead to the suspicion that the overall reduced laying performance of free-range flocks may be attributed to the non-synchronised peak performance of the various sub-populations rather than the housing system as such. Bodyweight, laying hen production performance and egg quality are essential key parameters for flock management and decision making. The data presented allows for a better understanding of these key factors in association with range usage. Significant differences of these sub-populations were observed for many parameters and can be used to explain the impact on the overall flock average, which is commonly monitored [21]. For example, a delayed onset and peak of lay as frequently observed in free-range flocks is most likely associated with the cohort of early stayers, while a drop in overall egg performance at the end of lay can be associated with the percentage of early rangers. Icken et al. (2008) [22] had previously investigated winter garden usage and daily nest box usage for nearly one year and detected a low negative correlation between laying performance and the frequency as well as the duration of winter garden use. The authors suggested that hens that often accessed the winter garden area may have not returned to the nest boxes for laying, but unfortunately, the authors did not collect floor eggs and as such, we are unable to conclude as to why a different outcome was observed in the present study. With every free-range farm housing a certain percentage of stayer or rangers based on the given circumstances (breed, shed design, pop hole size, flock size, geological region, etc.), individual management decisions of shed and range designs can be modified to increase or decrease the range use of the hens and potentially alter the impact that this may have on egg performance and size. While the sub-populations identified could be clearly distinguished at the beginning of the laying period, stayers increased the duration of their range visits over time (Figure 5). This is not surprising, as increased range use has been reported in older flocks and was suggested to be most likely accompanied with hen experience, maturity and familiarity of the environment [23]. The increased range usage contributed to a reduced variation among the replicates of stayers and rangers from 18 to 32 week of age. In the present study, early range usage was accompanied with a change of body weight, where both stayers and roamers were significantly lighter at point of lay compared to rangers at 16 and 22 weeks of age, but had comparable body weight at the end of lay. Inadequate bodyweight may have resulted in stayers preferring to spend time close to the feeder chains to meet the needs for physiological and metabolic requirements, but may have also impacted the social status of the hens [24]. For example, while range use can be related to the hens’ physiological ability and spatial memory, it has also been shown that hens that prefer to stay in the shed are consistently more fearful compared to ranging birds and have higher blood cortisol levels [7,25,26,27]. This association of fearfulness and increased blood cortisol levels in stayers may have also been a reason for the lower egg performance [25,27]. While the early stayers had an average body weight below breed standard [28], this would likely be the reason for the reduced egg performance with smaller eggs produced at 22 weeks of age [29]. Whether it was the increase in body weight which may then have triggered egg production or the increase of range use and the accompanying UV light exposure or an interaction thereof can only be speculated [30,31]. The range use of early stayers increased continuously and as such egg production of the stayers increased significantly as well. Despite the exposure to UV light, fibre/pasture intake is known to improve body condition development mediated by a stimulated gizzard function, intestinal organ development and subsequently nutrient digestibility [32,33,34].

Evidence has been gathered for decades showing how body weight as well as UV-light is essential for improved egg production and can be used in management decisions to manipulate egg outputs including egg quality [35,36,37], leading evidently to the question: is it the range use that promotes hen development and subsequently, laying performance, or is it hen development which allows hens to use the range while increasing their ovulation rate at the same time? Interestingly, egg production dropped significantly when birds were 52 weeks of age in all sub-populations, which is most likely associated to disease impact, diagnosed in flock A. It appears as if the disease affected early rangers more than the early stayers as shown by the 6.3% and 3.7% drop in egg production (Table 1). To allow further insight into the health challenges that the different sub-populations experienced, information about hen mortality and necropsy findings will be most useful and is currently under investigation.

The decreased egg production of the early rangers at the end of lay is concerning when considering the industry’s target of an extended flock life beyond 100 weeks of age [38]. Based on the data collected, it appears that for the current circumstances where laying periods frequently end at 80 weeks of age, early rangers would be the preferred hens of choice and question needs to be raised how the sub-population of early stayers in a shed can be minimised. However, when considering increased demands of the industry to extend the longevity of a flock, rangers seem currently not to be able to cope with the ongoing egg output [38]. Further research about the impact of early range use at end of lay performance would be highly warranted to allow for informed decision making regarding extended flock life. A first attempt to investigate the metabolic needs of stayers and rangers was performed by Kolakshyapati et al., 2020 [39]. Selecting commercial laying hens based on their range usage during 18–74 weeks of age and measuring their metabolic energy in closed-circuit calorimetry chambers, it became evident that light stayers had significantly higher metabolisable energy (ME) intake (*p* = 0.025), heat production (*p* = 0.005), and heat increment/body weight^0.75^ (*p* = 0.005) compared to light rangers. The higher maintenance energy required for light stayers may be of overall disadvantage and inefficient feed energy use that might be worth considering.

There was no effect of range use on albumen height, and yolk colour. Other investigators have seen darker egg yolks in ranging hens, most likely due to the increased uptake in plant canthaxanthins and carotenoids [40,41,42,43]. The differences of these studies compared to our results are most likely due to the use of in-feed pigments that the egg producer has adopted to ensure constant yolk colour regardless the potential pasture uptake. In addition, the average hen subjected to this research spent <1 h/day on the range, and it is likely that they did not forage for the entire duration of this ranging time [44]. Albumen height and Haugh unit decreased with the age of the hens, similar to Silversides and Budgell, 2004; Roberts, 2004, [45,46] where the highest Haugh unit is usually observed in hens at 22 weeks of age and gradually decreases with age. These results are similar to Matthews and Sumner, 2014 [47] who found that albumen height decreased with age regardless of the housing system (cage and free-range) from 25 to 60 weeks of age. These lack of relevant differences on egg quality between stayers and rangers indicates that there would be a limited disadvantage of housing rangers-only or stayers-only and that planning strategies do not need to consider egg size and their marketing when encouraging hens to range more or less.

Another impact of hen age could be observed on the percentage of system eggs obtained. The older the hens became, the fewer eggs were collected from the aviary system. This trend could be a result of transitioning the birds which may became more accustomed to using the nest box over time. Interestingly, stayers laid significantly more eggs in the system even though they would be in close physical proximity to the nest boxes (Table 3). Non-nest eggs increase the amount of labour required for egg collection while simultaneously reduce egg cleanliness and increase the number of eggs damaged or eaten by hens [48,49,50,51]. It might be, once again, the reluctance of these stayers to explore the environment that hindered those hens’ ability to use the nest boxes and highlights the impact that system-to-system rearing has on later use of resources. Also training hens to adapt good movement patterns by manually moving them off the floor at night or gently pushing them down from the top of the aviary system could potentially contribute to reduced system eggs [51,52,53]. Future research could investigate the area of the aviary system the system from which the system eggs were collected (e.g., top tier, bottom tier or other areas) and this could benefit farms that have a relatively high percentage of system eggs they need to manage. 

## 5. Conclusions

Stayers increased their range usage with age, while the hens that initially preferred to use the range were consistent in their ranging behaviour. While the effect of range use on egg performance was significant, the impact on egg grade and egg quality was minor. Managing the range use of sub-populations from an early age allows for informed decision-making and proactive control of the laying performance until the end of lay which is especially crucial when considering extending the end of lay beyond 100 weeks of age.

## Figures and Tables

**Table 1:** Tukey post-hoc comparison of the differences between stayers, roamers and rangers with respect to body weight and laying performance.

	Age of Hens (Weeks)	Stayers	Roamers	Rangers
**Bodyweight (kg)**	**16**	1.29 ± 0.021 ^h^	1.30 ± 0.022 ^g^	1.33 ± 0.021 ^f^
	**22**	1.68 ± 0.012 ^e^	1.74 ± 0.008 ^d^	1.78 ± 0.010 ^c^
	**74**	1.86 ± 0.020 ^b^	1.88 ± 0.031 ^a^	1.89 ± 0.027 ^a^
***p*** **-value Sub-population**		0.0001		
***p*** **-value Age of hens**		0.0001		
***p*** **-value Sub-population × Age**		0.0001		
**Laying performance (%)**	**22**	78.2 ± 1.9 ^Cb^	83.9 ± 1.6 ^Bab^	88.0 ± 1.1 ^ABa^
	**32**	93.5 ± 1.1 ^Aa^	86.1 ± 1.5 ^Bb^	91.6 ± 1.0 ^Aab^
	**42**	90.8 ± 2.3 ^ABa^	91.7 ± 0.8 ^ABa^	93.9 ± 0.7 ^Aa^
	**52**	87.1 ± 1.8 ^Ba^	83.8 ± 2.3 ^Ba^	87.3 ± 1.9 ^ABa^
	**62**	89.7 ± 3.0 ^ABa^	94.6 ± 1.3 ^Aa^	85.2 ± 1.5 ^Bb^
	**72**	95.5 ± 0.9 ^Aa^	89.9 ± 1.4 ^ABab^	85.1 ± 0.9 ^Bb^
***p*** **-value Sub-population**		0.3634		
***p*** **-value Age of hens**		0.0001		
***p*** **-value Sub-population × Age**		0.0001		

^ABCD^ Shows the significant difference of the values presented in the columns and values with a common letter on the same column do not differ significantly. ^abcd^ Shows the significant difference of the values presented in the rows and values with a common letter on the same row do not differ significantly.

**Table 2:** The difference between the egg quality of stayers, roamers and rangers at 22, 32, 42, 52, 62 and 72 weeks of age.

	Age of Hens (Weeks)	22	32	42	52	62	72
**Albumen height (mm)**	**Stayers**	7.48 ± 0.12 ^Aa^	7.20 ± 0.13 ^Aa^	7.17 ± 0.20 ^Aab^	5.83 ± 0.17 ^Bb^	6.30 ± 0.21 ^Ab^	5.8 ± 0.33 ^Ab^
	**Roamers**	7.47 ± 0.12 ^Aa^	7.36 ± 0.21 ^Aab^	6.49 ± 0.16 ^Aab^	5.61 ± 0.23 ^Bb^	5.75 ± 0.18 ^Ab^	5.36 ± 0.24 ^Ab^
	**Rangers**	7.76 ± 0.18 ^Aa^	7.32 ± 0.14 ^Aab^	6.70 ± 0.17 ^Aab^	7.40 ± 1.53 ^Aab^	5.41 ± 0.17 ^Ab^	5.56 ± 0.25 ^Ab^
***p*** **-value Sub-population**		0.128					
***p*** **-value Age of hen**		0.001					
***p*** **-value Sub-population × Age**		0.017					
**Egg weight (g)**	**Stayers**	51.3 ± 0.40 ^Ab^	70.7 ± 7.64 ^Aa^	64.3 ± 0.47 ^Aa^	62.0 ± 0.61 ^Aa^	61.8 ± 0.50 ^Aa^	64.6 ± 1.05 ^Aa^
	**Roamers**	51.5 ± 0.44 ^Ab^	57.1 ± 0.62 ^Aab^	63.3 ± 0.54 ^Aab^	62.0 ± 0.74 ^Aab^	69.8 ± 8.23 ^Aa^	62.4 ± 0.67 ^Aab^
	**Rangers**	51.7 ± 0.36 ^Aa^	60.5 ± 0.54 ^Aa^	63.9 ± 0.67 ^Aa^	61.6 ± 0.63 ^Aa^	62.0 ± 0.64 ^Aa^	61.4 ± 0.93 ^Aa^
***p*** **-value Sub-population**		0.5189					
***p*** **-value Age of hen**		0.0001					
***p*** **-value Sub-population × Age**		0.1246					
**Yolk colour**	**Stayers**	11.6 ± 0.10 ^Aab^	11.3 ± 0.17 ^Ac^	11.5 ± 0.11^A a^	11.9 ± 0.16 ^Aa^	10.9 ± 0.18 ^Acd^	10.6 ± 0.11 ^Ad^
	**Roamers**	11.5 ± 0.11 ^Ab^	11.8 ± 0.17 ^Aa^	11.6 ± 0.12 ^Ab^	11.9 ± 0.17 ^Aa^	10.8 ± 0.18 ^Ac^	10.2 ± 0.22 ^Ad^
	**Rangers**	11.4 ± 0.11 ^Ab^	11.8 ± 0.19 ^Ab^	11.5 ± 0.11^Ab^	12.3 ± 0.18 ^Aa^	10.9 ± 0.17 ^Ad^	10.6 ± 0.11 ^Ad^
***p*** **-value Sub-population**		0.4178					
***p*** **-value Age of hen**		0.0001					
***p*** **-value Sub-population × Age**		0.0939					
**Haugh unit**	**Stayers**	88.6 ± 0.62 ^Aa^	84.4 ± 0.77 ^Aab^	82.2 ± 1.36 ^Ab^	78.7 ± 0.97 ^Abc^	76.9 ± 1.44 ^Abc^	71.9 ± 2.42 ^Ac^
	**Roamers**	88.4 ± 0.70 ^Aa^	85.9 ± 0.97 ^aA^	79.6 ± 1.20 ^Ac^	73.7 ± 2.43 ^Ade^	73.1 ± 1.24 ^Ad^	69.9 ± 1.92 ^Ae^
	**Rangers**	89.5 ± 0.85 ^Aa^	85 ± 0.84 ^abA^	79.9 ± 1.27 ^Ab^	77 ± 1.71 ^Abc^	72.1 ± 1.21 ^Ac^	71.6 ± 1.88 ^Ac^
***p*** **-value Sub-population**		0.024					
***p*** **-value Age of hen**		0.0001					
***p*** **-value Sub-population × Age**		0.1013					
**Egg shell breaking strength (N)**	**Stayers**	4.53 ± 0.48 ^Aa^	4.54 ± 0.14 ^Aa^	4.9 ± 0.15 ^Ba^	4.62 ± 0.13 ^Aa^	4.12 ± 0.10 ^Aa^	4.11 ± 0.21 ^Aa^
	**Roamers**	4.18 ± 0.13 ^Ab^	4.95 ± 0.76 ^Aab^	6.47 ± 1.05 ^Aa^	5.28 ± 0.63 ^Aab^	4.05 ± 0.15 ^Ab^	3.76 ± 0.19 ^Aab^
	**Rangers**	4.42 ± 0.12 ^Aa^	4.24 ± 0.16 ^Aa^	4.38 ± 0.15 ^Ba^	4.5 ± 0.16 ^Aa^	4.51 ± 0.12 ^Aa^	3.81 ± 0.18 ^Aa^
***p*** **-value Sub-population**		0.1995					
***p*** **-value Age of hen**		0.0099					
***p*** **-value Sub-population × Age**		0.122					

^ABCD^ Shows the significant difference of the values presented in the columns and values with a common letter on the same column do not differ significantly. ^abcd^ Shows the significant difference of the values presented in the rows and values with a common letter on the same row do not differ significantly.

**Table 3:** Mean (±SEM) values of system, floor and waste eggs collected from the different stayers, roamers and ranger sub-populations at 22, 32, 42, 52, 62 and 72 weeks of age.

	Age of Hens (Weeks)	22	32	42	52	62	72
**System eggs (%)**	**Stayers**	3.24 ± 0.37 ^Aa^	2.20 ± 0.26 ^Ab^	1.31 ± 0.30 ^Abc^	1.13 ± 0.16 ^Ac^	0.58 ± 0.13 ^Ac^	0.29 ± 0.07 ^Ad^
	**Roamers**	3.11 ± 0.30 ^Aa^	1.58 ± 0.17 ^Bb^	0.91 ± 0.19 ^Bbc^	0.87 ± 0.12 ^ABbc^	0.60 ± 0.13 ^Ac^	0.55 ± 0.13 ^Ac^
	**Rangers**	2.77 ± 0.21 ^Aa^	1.18 ± 0.13 ^Bb^	0.55 ± 0.11 ^Bbc^	0.67 ± 0.09 ^Bbc^	0.63 ± 0.09 ^Abc^	0.45 ± 0.09 ^Ac^
***p*** **-value Sub-population**		0.0001					
***p*** **-value Age of hen**		0.0001					
***p*** **-values Sub-population × Age**		0.0177					
**Floor eggs (%)**	**Stayers**	0.43 ± 0.08 ^Aa^	0.34 ± 0.06 ^Aa^	0.30 ± 0.09 ^Aa^	0.38 ± 0.06 ^Aa^	0.54 ± 0.14 ^Aa^	0.36 ± 0.05 ^Aa^
	**Roamers**	0.54 ± 0.10 ^Aa^	0.61 ± 0.08 ^Aa^	0.55 ± 0.08 ^Aa^	0.43 ± 0.07 ^Aa^	0.43 ± 0.11 ^Aa^	0.46 ± 0.08 ^Aa^
	**Rangers**	0.40 ± 0.06 ^Aa^	0.40 ± 0.07 ^Aa^	0.41 ± 0.06 ^Aa^	0.42 ± 0.09 ^Aa^	0.33 ± 0.04 ^Aa^	0.48 ± 0.07 ^Aa^
***p*** **-value Sub-population**		0.0148					
***p*** **-value Age of hen**		0.5796					
***p*** **-value Sub-population × Age**		0.3987					
**Waste eggs (%)**	**Stayers**	0.07 ± 0.04 ^Aa^	0.14 ± 0.04 ^Aa^	0.05 ± 0.03 ^Aa^	0.32 ± 0.09 ^Aa^	0.32 ± 0.13 ^Aa^	0.35 ± 0.07 ^Aa^
	**Roamers**	0.29 ± 0.10 ^Aa^	0.07 ± 0.02 ^Ab^	0.11 ± 0.05 ^Ab^	0.21 ± 0.06 ^Ab^	0.21 ± 0.06 ^Ab^	0.57 ± 0.07 ^Aa^
	**Rangers**	0.09 ± 0.04 ^Ab^	0.11 ± 0.04 ^Ab^	0.05 ± 0.02 ^Ab^	0.11 ± 0.04 ^Ab^	0.22 ± 0.04 ^Ab^	0.50 ± 0.09 ^Aa^
***p*** **-value Sub-population**		0.2049					
***p*** **-value Age of hen**		0.0001					
***p*** **-value Sub-population × Age**		0.0354					

^ABCD^ Shows the significant difference of the values presented in the columns and values with a common letter on the same column do not differ significantly. ^abcd^ Shows the significant difference of the values presented in the rows and values with a common letter on the same row do not differ significantly.

**Table 4:** The difference between the stayers, roamers and rangers on different egg grades at 22, 32, 42, 52, 62 and 72 weeks of age.

	Age of Hens (Weeks)	22	32	42	52	62	72
**Jumbo eggs [66.7–78 g] (%)**	**Stayers**	1.94 ± 0.14 ^Ac^	19.7 ± 3.68 ^Aa^	8.16 ± 0.93 ^Abc^	9.44 ± 0.78 ^Abc^	11.4 ± 0.52 ^Ab^	13.7 ± 0.66 ^Aab^
	**Roamers**	1.98 ± 0.13 ^Ac^	21.4 ± 3.59 ^Aa^	8.31 ± 1.01 ^Acd^	9.17 ± 1.16 ^Acd^	10.4 ± 0.68 ^Ac^	11.5 ± 0.42 ^Ac^
	**Rangers**	2.30 ± 0.16 ^Ac^	21.7 ± 3.65 ^Aa^	7.03 ± 0.70 ^Abc^	8.80 ± 0.99 ^Abc^	8.50 ± 0.60 ^Abc^	11.5 ± 0.47 ^Ab^
***p*** **-value Sub-population**		0.6396					
***p*** **-value Age of hen**		0.0001					
***p*** **-value Sub-population × Age**		0.9609					
**X-large eggs [58.3–66.6 g] (%)**	**Stayers**	4.16 ± 1.33 ^Ad^	48.1 ± 3.16 ^Ac^	69.7 ± 0.67 ^Aa^	65.3 ± 1.60 ^Aab^	65.5 ± 1.15 ^Aab^	60.4 ± 1.13 ^Ab^
	**Roamers**	2.94 ± 0.21 ^Ad^	46.3 ± 3.31 ^Ac^	70.5 ± 1.03 ^Aa^	61.1 ± 1.89 ^Aab^	66.6 ± 0.83 ^Aab^	59.9 ± 1.23 ^Ab^
	**Rangers**	3.27 ± 0.21 ^Ad^	45.7 ± 2.75 ^Ac^	70.7 ± 1.08 ^Aa^	64.2 ± 1.52 ^Aab^	67.4 ± 2.30 ^Aab^	60.7 ± 1.18 ^Ab^
***p*** **-value Sub-population**		0.5846					
***p*** **-value Age of hen**		0.0001					
***p*** **-value Sub-population × Age**		0.9204					
**Large eggs [50–58.2 g] (%)**	**Stayers**	38.9 ± 1.28 ^Ba^	26.1 ± 1.70 ^Ab^	17.2 ± 1.82 ^Abc^	16.4 ± 1.75 ^Abc^	14.9 ± 0.66 ^Abc^	10.4 ± 0.73 ^Ac^
	**Roamers**	45.2 ± 1.54 ^Aa^	26.5 ± 1.28 ^Ab^	17.0 ± 1.79 ^Abc^	16.8 ± 1.56 ^Abc^	15.8 ± 0.40 ^Abc^	10.3 ± 0.88 ^Ac^
	**Rangers**	49.2 ± 1.48 ^Aa^	27.3 ± 1.56 ^Ab^	17.0 ± 1.99 ^Abc^	17.6 ± 1.89 ^Abc^	13.8 ± 0.85 ^Abc^	12.0 ± 0.60 ^Abc^
***p*** **-value Sub-population**		0.0198					
***p*** **-value Age of hen**		0.0001					
***p*** **-value Sub-population × Age**		0.0046					
**Medium eggs [42–49.9 g] (%)**	**Stayers**	46.1 ± 1.56 ^Aa^	0.30 ± 0.09 ^Ab^	0.19 ± 0.04 ^Ab^	0.47 ± 0.13 ^Ab^	0.30 ± 0.06 ^Ab^	0.01 ± 0.01 ^Ab^
	**Roamers**	44.4 ± 1.43 ^Aa^	0.56 ± 0.18 ^Ab^	0.24 ± 0.07 ^Ab^	0.47 ± 0.14 ^Ab^	0.08 ± 0.03 ^Ab^	0.09 ± 0.03 ^Ab^
	**Rangers**	38.0 ± 1.37 ^Ba^	0.38 ± 0.11^A b^	0.17 ± 0.07 ^Ab^	0.55 ± 0.13 ^Ab^	0.17 ± 0.04 ^Ab^	0.15 ± 0.04 ^Ab^
***p*** **-value Sub-population**		0.0028					
***p*** **-value Age of hen**		0.0001					
***p*** **-value Sub-population × Age**		0.0001					
**Economy eggs [<42 g] (%)**	**Stayers**	1.79 ± 0.28 ^Aa^	0.00 ± 0.00 ^Ab^	0.02 ± 0.02 ^Ab^	0.04 ± 0.02 ^Ab^	0.04 ± 0.02 ^Ab^	0.03 ± 0.02 ^Ab^
	**Roamers**	1.19 ± 0.12 ^Ba^	0.02 ± 0.01 ^Ab^	0.03 ± 0.02 ^Ab^	0.02 ± 0.02 ^Ab^	0.01 ± 0.01 ^Ab^	0.00 ± 0.00 ^Ab^
	**Rangers**	0.99 ± 0.11 ^Ba^	0.05 ± 0.03 ^Ab^	0.04 ± 0.02 ^Ab^	0.06 ± 0.03 ^Ab^	0.07 ± 0.03 ^Ab^	0.15 ± 0.03 ^Ab^
***p*** **-value Sub-population**		0.0707					
***p*** **-value Age of hen**		0.0001					
***p*** **-value Sub-population × Age**		0.0001					
**Pulp (%)**	**Stayers**	7.19 ± 0.77 ^Ac^	5.87 ± 0.84 ^Ac^	4.74 ± 0.89 ^Ac^	8.42 ± 1.27 ^Ab^	7.79 ± 1.02 ^Ab^	15.5 ± 1.33 ^Aa^
	**Roamers**	4.32 ± 0.49 ^Ac^	5.25 ± 0.56 ^Ac^	3.91 ± 0.73 ^Ac^	12.4 ± 1.87 ^Aab^	7.19 ± 0.69 ^Ab^	18.3 ± 1.75 ^Aa^
	**Rangers**	6.25 ± 1.46 ^Ac^	4.94 ± 0.52 ^Ac^	5.01 ± 0.82 ^Ac^	8.81 ± 1.21 ^Abc^	10.1 ± 2.92 ^Ab^	15.6 ± 1.25 ^Aa^
***p*** **-value Sub-population**		0.9193					
***p*** **-value Age of hen**		0.0001					
***p*** **-value Sub-population × Age**		0.1005					

^ABCD^ Shows the significant difference of the values presented in the columns and values with a common letter on the same column do not differ significantly. ^abcd^ Shows the significant difference of the values presented in the rows and values with a common letter on the same row do not differ significantly.
